# Breastfeeding experience of postnatal mothers separated from preterm infants after discharge: a phenomenology qualitative approach

**DOI:** 10.1186/s12884-023-06230-z

**Published:** 2024-01-04

**Authors:** Xin Jiang, Hui Jiang, Shan Shan Shan, Rong Huang

**Affiliations:** Shanghai First Maternity and Infant Hospital, School of Medicine, Tongji University, Shanghai, 200092 China

**Keywords:** Maternal-infant separation, Preterm infant, Breastfeeding challenges, Breastfeeding support, Qualitative study

## Abstract

**Background:**

Breastfeeding practices are influenced by the maternal-infant bond relationship. Mothers of preterm infants in the puerperium face many challenges and support is needed to maintain breastfeeding after hospital discharge. This study explored the breastfeeding experiences among mothers of preterm infants and challenges that influenced their breastfeeding practices.

**Methods:**

A qualitative phenomenological approach was used involving the mothers of preterm infants during the puerperium in Shanghai who fulfilled the inclusion criteria and consented to participate in the study. The mothers were recruited using purposive sampling. Eighteen participants were interviewed using semi-structured in-depth interviews. All interviews were recorded in digital audio, transcribed verbatim, and analyzed using thematic analysis.

**Findings:**

The breastfeeding experience among mothers of preterm infants included four themes: breastfeeding motivation, breastfeeding challenges, breastfeeding support and education, and response to parental stress. Breastfeeding challenges included perceived insufficient milk, bottle preference, and maternal-infant separation. Two sub-themes of breastfeeding support included breastfeeding knowledge and approach.

**Conclusion:**

To overcome breastfeeding challenges and improve the breastfeeding rate of preterm infants after discharge, medical professionals must develop individualized breastfeeding plans based on a comprehensive assessment of the needs of mothers who delivered a preterm infant.

**Supplementary Information:**

The online version contains supplementary material available at 10.1186/s12884-023-06230-z.

## Background

Preterm birth (delivery before 37 weeks gestation) has received inadequate attention and funding, in part to a lack of information on the scope of the issue globally. A recent report from the US March of Dimes Foundation [1], which was based on a statistical analysis from the World Health Organization (WHO), attempted to resolve this issue. The prevalence of preterm births was estimated both regionally and globally in the report. The number or preterm births is troubling. Specifically, an estimated 13.4 million infants were born preterm in 2020. Approximately 900,000 infants died of preterm birth complications in 2019. Many preterm birth survivors face a lifetime of disability, including learning disabilities, and visual and hearing impairment [2]. Historically, preterm infants admitted to neonatal units have encountered policies and practices that are barriers to successful breastfeeding, such as delayed initiation of breastfeeding, mother-infant separation, and bottle feeding [3].

Breastfeeding is associated with a lower risk of acute and chronic diseases, such as late-onset sepsis, necrotizing enterocolitis, and ventilator-associated pneumonia, all of which are issues for small, ill, and preterm infants [4, 5]. Prematurity, maternal-infant separation, and co-morbidities are well-known breastfeeding obstacles [6].

The Baby-friendly Hospital Initiative (BFHI) was launched by the WHO and the United Nations Children’s Fund (UNICEF) in 2020 for preterm infants [7, 8]. The BFHI promotes the benefits of breastfeeding and encourages maternal breastfeeding behavior.

There is substantial evidence that the BFHI Ten Steps has been implemented in developed and developing countries. In the USA [9] and Canada [10], higher breastfeeding rates have more likely resulted from being tied to institutional support and a robust infrastructure, including written breastfeeding policies [11]. Facilities in the USA that update breastfeeding policies have higher breastfeeding rates [12]. Indeed, breastfeeding promotion in the USA is positively impacted by the transition from written breastfeeding and infant feeding guidelines to a formal signed breastfeeding policy in most maternity units [13]. The importance of administrative staff commitment to the BFHI, receiving facility accreditation, and offering ongoing assistance to parturients has been emphasized in Australia [14]. A “hearts and minds” strategy has been deemed helpful to implement the WHO/UNICEF BFHI in UK communities [15]. Investment in the BFHI Ten Steps in a Surabaya maternity facility (Indonesia) produced a social value 49 times greater than the cost of investment [16]. Data from hospitals in China suggest that higher compliance with baby-friendly practices may have a positive impact on exclusive breastfeeding (EBF) at 3 months [17]. There is mounting evidence that standard BFHI designation has a spill over effect with improved human milk use in the hospital’s neonatal ward in the developed and developing countries. however, the current state of preterm infant breastfeeding is not encouraged worldwide. The in-hospital breastfeeding rate of preterm infants in developed countries ranges from 13 to 49% [18, 19].

Based on an in-depth study and ongoing research involving international BHFIs, a preliminary policy system has been formed, a team of experts in the field has been organized, and practical methods, such as co-location of mothers and newborns in the same room have, been explored and popularized throughout China [20]. Even though policies that promote breastfeeding in preterm infants have been implemented in China [21, 22], the breastfeeding rate is still only 15% for preterm infant in hospitals [22]. This finding indicates that the current state of breastfeeding in preterm infants who have been separated from their mother during hospitalization is not ideal; much less is reported due to drop-outs and loss to follow-up.

Mothers who are separated from their preterm infants are more likely to experience breastfeeding failure. Negative attitudes toward breastfeeding predominate because mothers who deliver a preterm infant encounter numerous breastfeeding difficulties when separated from their preterm infants [23, 24]. The current qualitative study was conducted to explore breastfeeding experiences among mothers who were separated from their preterm infants post-delivery in Shanghai, China.

## Methods

### Design

A qualitative phenomenological approach was used to explore the breastfeeding experiences among the mothers of preterm infants. Semi-structured in-depth interviews were used to discuss breastfeeding experiences encompassing issues related to separation that were deemed challenging by the mothers of preterm infants. The results were reported using the Consolidated Criteria for Reporting Qualitative Research (COREQ [see Additional file 1]) [25].

### Participants and sampling

The researchers made daily rounds on the postpartum ward asking the nurses if there were any parturients who met the inclusion and exclusion criteria. The mothers who agreed to be enrolled were informed in the hospital that they would receive a face-to-face interview in the outpatient clinic at the 42-day postpartum follow-up visit. Before the research officially began, we created an operation manual to standardize all study procedures, identify potential problems, and address any issues in the pilot trial.

From January to July 2019, a purposive sampling approach was used and 18 mothers of preterm infants who fulfilled the inclusion and exclusion criteria after hospital discharge were included as study participants for semi-structured interviews. Six parturients with breastfeeding experience and 12 parturients without breastfeeding experience were recruited for the study.

Based on the literature review and clinical experience, the inclusion criteria for the sample were as follows: gestational age < 37 weeks at the time of delivery, ability to read and communicate, provide informed consent and voluntarily agree to participate in the research, and preterm infants admitted to the neonatal intensive care unit (NICU). The exclusion criteria were as follows: primiparas who used oral sleeping pills, a history of mental illness, or declining to breastfeed.(Because the primiparous mothers on medication are at high risk of breastfeeding failure ,so they were excluded).Data were gathered until the theoretical saturation point was reached, which occurred after 18 interviews. Data saturation refers to the point in the research process when no new information is discovered in data analysis and further data collection would yield similar results [26]. In addition, data saturation is the point at which researchers stop collecting data [27]. In the current study, no additional information surfaced after the 16th mother was questioned, therefore two additional mothers were interviewed to assure data saturation. No participants withdrew from the study.

Study participants were recruited from the mothers of infants who had been born preterm and hospitalized in the NICU at Shanghai First Maternity and Infant Hospital, a Tongji University-affiliated tertiary A-grade hospital in China that specializes in obstetrics and gynecology. Shanghai First Maternity and Infant Hospital implemented the BFHI in 1992. Potential parturients were informed about the study during their hospitalization, and that study participants would have a face-to-face interview in the Outpatient Clinic at the 42-day postpartum follow-up visit.

The researcher invited the mothers of three preterm infants who met the inclusion criteria to participate in pilot interviews to assess the feasibility, clarity, and appropriateness of the interview questions and improvements were made accordingly. The interview guide was tested by the two authors in three pilot interviews, which resulted in no changes.

Before the interview, the researcher described the purpose of the study to the potential participants, had an informed consent form signed, and set up a convenient time and location for the face-to-face interview. Then, the official interviews began. The interviews were recorded in digital audio using a smartphone recorder.

### Researchers and positionality

All team members were female registered nurses with backgrounds in breastfeeding research and descriptive qualitative research experience. All interviews were conducted by the first author (JX), who is working towards a medical degree. JX had one child and had a positive breastfeeding experience; she claimed no biases for or against breastfeeding. HR had a son who was 2-years-old at the time the study began and had a positive breastfeeding experience. SSS had a bachelor’s degree in nursing. SSS had a son who was 10-years-old at the time the study began. HJ, a skilled qualitative researcher with an adult child, frequently offered expert methodologic support.

### Ethics consideration

Prior to conducting the study, the Ethics Committee of Shanghai First Maternity and Infant Hospital granted approval (No. KS23313). The tenets of the Declaration of Helsinki were followed. The goals and confidentiality of the study were made clear to each participant. Each participant signed the informed consent and their interview recorded. Confidentiality was upheld by concealing participant names or private information. The audio recordings and transcripts were all password-protected, coded, and saved on a computer. Mothers who agreed to participate were free to depart at any time without any consequences.

### Data collection

Each in-depth semi-structured interview was conducted in a quiet clinic room with only the study participant and interviewer present. Relevant socio-demographic respondent data were collected prior to conducting the interviews.

An interview guide was developed by the research group and consisted of six open-ended questions, as follows: “How is your breastfeeding progressing so far?” “How long you plan to breastfeed?” “What breastfeeding issues, if any, have you encountered?“ “What method did you use to solve breastfeeding issues?“ “What kind of support and assistance did you receive while breastfeeding? “ “What are the most pressing breastfeeding issues you encountered?“ The interview lasted 40 − 50 min, depending on the participants’ willingness to provide details about their experiences. The entire interview was recorded on a cell phone. Each interview was followed by a quick note-taking session in the field.

In addition to the information conveyed verbally, non-verbal expressions and behaviors were also observed during the interviews. Non-verbal communication has been acknowledged as a powerful informational tool and as a complement to research on human verbal actions [28]. Non-verbal communications were observed and evaluated on video recordings. To minimize potential observer bias, our teams followed the following steps: (1) use of masking or blinding helped us assure that our participants and observers were unaware of the research aims. This procedure removed research expectations by knowing the study purpose, so the observers are less likely to be biased. (2) With multiple observers in the study, we were able to ensure that the data were consistent and not biased by the bias of one observer. (3) We trained all observers to confirm everyone collects and records data per protocol. (4) We created standardized procedures or protocols that were structured and easy to understand for all observers. (5) Participants were asked to reconfirm the results of the interviews. The researcher refined the thematic framework according to the information provided by the interviewees in a specific order, so that the final themes were intrinsically relevant.

### Data analysis

Each interview was conducted in Mandarin and within 24 − 48 h of each interview the tapes were transcribed verbatim for coding. The transcripts were returned to 12 participants for review; the other six participants declined to carefully read the transcripts due to busy schedules. No errors or adjustments were made by the participants who read the transcripts. Computer-assisted qualitative data analysis software (CAQDAS) NVivo 12 was used to maintain and analyze transcripts [29]. In the event of a disagreement, a third author was invited to arrive at a consensus on the final coding choice after two writers had coded the data. The Colaizzi seven-step phenomenologic analysis method was utilized to determine themes and sub-themes [30]. This process follows the data closely and is rigorously analytical: (1) become familiar with the transcripts from the collected recordings carefully and repeatedly, (2) identify pertinent, significant, and meaningful utterances, (3) determine meanings for recurring ideas and provide codes, (4) cluster initial themes by searching for common coded ideas, (5) describe the themes in detail and choose appropriate utterances as quotes, (6) create and redefine themes and subthemes, and (7) seek validation from others. The authors adjusted the final stage using feedback from the previous steps of the study. Double coding, member checking, bracketing, and peer debriefing were used to confirm study validity [31, 32].

The pertinent data were organized and systematized for the open research questions when the data were coded through a thematic analysis. The interviews were directly recorded by two authors. All the authors read the transcribed material numerous times to become comfortable with the content. The initial concepts of the data were noted and debated. The characteristics of the data that were relevant to the goal of the investigation were given initial codes. Equal attention was allotted to each interview, which was then coded for as many patterns as feasible. Then, two of the authors arranged the codes into themes, which were then collectively updated by the four authors. The initial topics were examined based on the complete body of information. To develop themes that were logical and consistent, coded excerpts were shifted. And to assist in the development of topics, thematic maps were developed. Finally, the themes were described by determining the central idea of each subject and outlining its content. Each author contributed to the process of clarifying the terms and assigning labels to the themes. Citations were selected to support the findings as the manuscript was being written.

### Findings

#### Demographic characteristics of the participants

The mothers of 18 preterm infants, 20–42 years of age, voluntarily participated in this study. All research participants were Chinese who attained at least a college level of education. Seven participants had spontaneous vaginal deliveries and 11 were delivered via cesarean section. Six parturients breastfed, but not exclusively. Twelve parturients were recruited who did not breastfeed. No participants withdrew from the study. 8 girls and 10 boys were delivered. The gestational ages ranged from 28 to 35 weeks. All preterm newborns were admitted to the NICU and the average length of hospital stay was 22.41 ± 9.03 days (Table 1).

**Table 1:** Demographic characteristics of research participants

ID	Age	Occupation	Delivery Mode	Education level	Baby Sex	Gestational Week	stay length	Breastfeeding experience
1	20	Teacher	SD	College	Male	28–28^+ 6^	32	No
2	24	Clerk	CD	undergraduate	Female	35–35^+ 6^	10	Yes
3	28	None	SD	College	Female	34–34^+ 6^	10	Yes
4	37	Manager	CD	undergraduate	Male	29–29^+ 6^	32	No
5	42	Clerk	CD	College	Female	34–34^+ 6^	12	Yes
6	40	None	CD	undergraduate	Male	34–34^+ 6^	8	Yes
7	34	Nurse	SD	College	Female	28–28^+ 6^	29	No
8	38	Doctor	CD	undergraduate	Male	31–31^+ 6^	18	No
9	26	Lawyer	SD	graduate	Male	28–28^+ 6^	35	No
10	25	Clerk	SD	graduate	Male	30–30^+ 6^	28	No
11	24	Teacher	CD	College	Male	31–31^+ 6^	22	No
12	28	Clerk	SD	undergraduate	Male	29–29^+ 6^	30	Yes
13	30	Saleswoman	SD	College	Female	30–30^+ 6^	25	No
14	32	Clerk	CD	undergraduate	Female	29–29^+ 6^	28	No
15	34	None	CD	Doctor	Male	32–32^+ 6^	30	No
16	36	Clerk	CD	College	Female	33–33^+ 6^	18	Yes
17	40	Baby sitter	CD	College	Male	33–33^+ 6^	14	No
18	29	Engineer	CD	undergraduate	Female	29–29^+ 6^	30	No

The current qualitative study identified four overarching themes from the breastfeeding experiences of mothers who had preterm infants after being discharged from the hospital using the Colaizzi seven-step pedagogic approach, as follows: motivation to breastfeed, breastfeeding challenges, breastfeeding support, and parental stress coping. Subthemes for some of the themes included breastfeeding challenges, which were further divided into three sub-themes (conscious lactation insufficiency, nipple confusion, and mother-infant separation. Breastfeeding knowledge support and breastfeeding method support were the sub-themes under breastfeeding support (Table 2).

**Table 2:** Themes and subthemes

Themes	Subthemes
Breastfeeding motivation	
Breastfeeding challenge	Perceived insufficient milk
	Nipple confusion/ bottle preference
	Maternal-infant separation
Breastfeeding support and education	Breastfeeding knowledge
	Breastfeedingapproach
Response to parental stress.	

### Theme 1: breastfeeding motivation

The mothers of the preterm infants gave several reasons for wanting to breastfeed and they all intended to persevere and achieve their goal of EBF. Mothers frequently cited a variety of physical benefits of human milk for the newborn, including dietary advantages, immunity, and growth. The NICU is an intricate and multifaceted care environment, with the NICU patient population among the most vulnerable in the hospital setting. The NICU is a challenging environment in which to initiate parenthood, and the negative effects on emotional wellness serve to highlight the impact of this experience on the lives of individuals and families. Parents cited a variety of contextual factors, including logistical factors (transportation, housing/accommodations close to the hospital, and access to affordable and nutritious food), relationship factors (trusting relationships with care providers, continuity of care [availability of primary nurses and physicians], and unbiased/non-judgmental care), and social-emotional factors (flexible visiting polices with limited restrictions accessing the infant). Due to the risk of infection in preterm newborns, the NICU is a closed environment where mothers and families cannot enter to look after their babies anytime, so they have to deliver breast milk to the NICU for bottle feeding, especially in China. Mothers and their preterm newborns were separated after birth, and mothers with insufficient milk reported a tremendous desire to parent and strengthen the intimate bond with their newborns through breastfeeding. All mothers of preterm infants expressed a need to learn breastfeeding techniques, but also wanted to understand infant cues and behaviors to optimize the breastfeeding experience. Many of the parturients stated that if their infants had not been preterm, they would have given up on trying to breastfeed. “If breastfeeding had been this difficult and she had been full term, I would have given up, but since she’s preterm, we push (participant 6).” Because the parturients thought their breastfeeding experience would be dramatically different when their newborns were discharged, mothers were encouraged to breastfeed during the puerperium. “My baby came home from the hospital and I wanted to breastfeed because I didn’t think I would go into the NICU, otherwise I would have breastfed after delivery (participant 3).” “I wished her to suckle my nipple, I guess it’s in the nature of newborns, and it would bring me closer to her (participant 5).” “I especially want to hold my daughter in my arms and breastfeed her like other mothers do, so I’m sure I’ll be breastfeeding her (participant 9.” “ I’ll keep on breastfeeding in the future, because I can’t constantly bottle feed her (participant 4).”

### Theme 2: breastfeeding challenge

Breastfeeding was affected by the following difficulties: perceived insufficient milk, bottle preference, and maternal-infant separation. These challenges negatively influenced breastfeeding according to the participants.

### Subtheme: perceived insufficient milk

The main challenge in breastfeeding for the mothers of preterm infants was feeling that their milk was inadequate when their babies were discharged from the hospital. The main reason for using formula was insufficient breast milk: “The baby had formula milk added when he was in the NICU; I still didn’t produce enough milk after he was discharged so I have to supplement formula (participant 11).”

### Subtheme: nipple confusion/ bottle preference

A mother of a preterm infant stated that she had an issue with bottle preference and the infant refused to latch onto her nipple. “My baby was admitted to the NICU because of premature birth, so he had been bottle-fed, and now my baby was discharged from the hospital, and my breast milk is also sufficient, but I find that the baby always cries when hungry, and not opening his mouth at all when he is full. How can I make my baby breastfeed (participant 7)?”

### Subtheme: maternal-infant separation

Separation of the mother and infant is the biggest challenge for breastfeeding preterm infants. Mother-infant separation interrupts the establishment of normal lactation after childbirth, which has a serious impact on the mother and infant. “When I wanted to breastfeed my baby, he was in the NICU and I couldn’t make it (participant 7).” “I didn’t hand-express the breast milk because the baby wasn’t with me. (participant 10).”

### Theme 2: breastfeeding support

#### Subtheme: breastfeeding knowledge

Support is a crucial aspect of breastfeeding. The mothers of preterm infants said they wanted information support, that they did not get sufficient breastfeeding education, such as breastfeeding-related knowledge during the ante- and post-partum, confusion and a lack of confidence during breastfeeding, and a higher demand for breastfeeding. “What should I do if my baby does not latch the nipple after being discharged from the hospital (participant 2)?” “What I know about breastfeeding is a concept; I hope you can give me more detailed guidance and help substantially (participant 1).” “Now my baby is back with me, but I haven’t received any training on breastfeeding. Can you give me some tips for breastfeeding (participant 8)?” “My baby was born prematurely and admitted to the NICU. Although I was told to express or pump breastmilk during the hospitalization, I still didn’t know how to breastfeed after my baby was discharged (participant 18).” “I still trust you guys; I hope you can have a hotline or an internet platform to make a consultation (participant 17).”

### Subtheme: breastfeeding approach

The mothers of preterm infants want to be provided expert counseling based on their individual circumstances, as well as a need to visualize or watch the breastfeeding process. “Do you have any videos or anything like that that I can see because textual things aren’t that intuitive or can you share the breastfeeding experience of mothers of preterm babies (participant 15)?” “I believe it is best for you to show me on site and provide one-on-one advice to solve problems (participant 13).” “I think it would be beneficial for you to establish a public breastfeeding consultant website online for preterm babies so that I’ll know how to deal with the situation if my baby is born prematurely (participant 14).”

### Theme 4: response to parental stress

Parenting stress is defined as the stress experienced by a father or mother within the parent-child bond. Therefore, the parents of preterm infants experience pressure resulting from their parenting responsibilities, as well as the social and environmental contexts in which the parents find themselves. In addition to coping with an infant’s serious illness and the uncertainty surrounding the long-term prognosis, parents with a newborn in the NICU frequently experience emotional and psychological stress from the hospitalization. Mothers of preterm infants believe that family members, especially fathers, are important and that active paternal participation in the care of the preterm infants, active learning about breastfeeding, and assisting in the breastfeeding process can reduce parenting stress among mothers, and thus promote breastfeeding. “It is always the father who hiccups the baby after breastfeeding (participant 16).” “My husband was important in strengthening my breastfeeding confidence and he always enlightened me (participant 9).” “He encourages me when I want to give up breastfeeding (participant 10).

## Discussion

This research aimed to explore the breastfeeding experiences of mothers separated from their preterm newborns after discharge from the hospital. The results focused on breastfeeding motivation, breastfeeding challenges, breastfeeding support and education, and response to parental stress.

Although the parturients acknowledged that breast milk was the best for their preterm infants, when a preterm newborn is admitted to the NICU, parents are authorized to visit once a week in the local hospital. Therefore, the parturients believed that separation adversely affected breastfeeding. Breastfeeding may be a challenge and due to the immature physiologic and neurodevelopmental systems of preterm infants, several problems occur. During the interviews we discovered that the mothers of preterm infants had a strong desire to breastfeed. EBF of preterm infants during the hospital stay is key to successful breastfeeding after hospital discharge [33]. The facility should prepare parents of perterm infants for continued breastfeeding and ensure access to support services/groups after hospital discharge [8]. The self-efficacy of mothers in breastfeeding is central to understanding which mothers are going to breastfeed their infants [34]. Persons with low self-efficacy often find tasks difficult to perform. If persons with low self-efficacy fail, they will blame themselves, whereas persons with high self-efficacy are prepared to test and try until they reach a solution. Based on the Feng study [35], we found that mothers of preterm infants reported physically and mentally challenging breastfeeding experiences during the period they were separated from their infants. Most mothers and family members of preterm infants lack breastfeeding knowledge and skills, especially expressing breastmilk and establishing lactation. With little professional support available, the mothers depend upon non-professionals to establish breastfeeding. This finding agrees with the findings of the current study. Therefore, the mothers of these preterm infants may need additional assistance in establishing and maintaining milk supply.

Second, mothers of preterm infants should be educated about the benefits of breastfeeding as well as the methods for collecting, storing, and delivering breast milk after delivery. Indeed, breastfeeding education should be given and bolstered through videos and knowledge questionnaires in the 3rd trimester of pregnancy, particularly in high risk obstetric clinics because mothers of preterm infants face the possibility of mother-infant separation [36]. Finally, among mothers of preterm infants early postpartum skin-to-skin contact and “latching on” were encouraged, and instructions were given on how to set lactation goals. A thorough examination was performed to determine the condition of the infant. After the infant was stabilized, a comprehensive assessment was performed, which included assessing the physical condition of the mother, lactation, and breastfeeding knowledge. Assessing the oral structure and sucking development of the preterm infant, assessing family member information, emotions, and behavioral support for breastfeeding, and developing an individualized breastfeeding program were completed.

Kangaroo care not only boosts mother lactation and increases parent-infant relationships [37], but also directly increases the breastfeeding rate [38]. For breastfeeding to succeed among preterm infants, hospital staff should focus on the individual mother and her situation, and the facility should provide family-centered care within a supportive environment, including kangaroo mother care for preterm infants [39]. In fact, kangaroo care should be initiated as early and frequently as possible to promote breastfeeding in preterm infants [40]. Maternal presence and early, frequent, prolonged, if not continuous, skin-to-skin care are essential for the mother of a preterm infant to learn her infant’s feeding and distress cues and respond appropriately. Involving the mother in the care of her infant gives the mother confidence in handling her child and reduces worry regarding the baby’s condition [39]. The care is initiated in the hospital and continued at home with adequate support and follow-up when discharged from the hospital early [8] .Based on a literature review, the most concerning issue for preterm infants is the ability to organize sucking patterns and the coordination of suck-swallow-breathe [41, 42]. Chen et al. [43] reported that oral feeding of preterm infants resulted in a considerable increase in transoral feeding. As a result, kangaroo care and integrated oral motor therapies for extremely preterm children are considered when developing personalized programs to enhance communication. As a result, in the creation of tailored programs to encourage breastfeeding in preterm infants, kangaroo care and integrated oral motor therapies for the preterm infants are considered.

The development of a breastfeeding support system necessitates a high level of medical skill, training updates, and adequate breastfeeding-related knowledge. Global studies have shown that breastfeeding support from trained healthcare professionals in conjunction with organizational support for breastfeeding are important variables for breastfeeding success [44]. Currently, breastfeeding practices for preterm infants in the NICU include the delivery of breastmilk, instruction in the collection, transfer, and use of breastmilk for preterm mothers, and regular training for all NICU medical staff on breastfeeding-related knowledge and norms. The WHO calls for use of donor human milk as the feeding of choice, if mother’s own milk is insufficient, unavailable or contraindicated [45]. Then, our breastfeeding team includes several internationally certified lactation consultants who teach breastfeeding specialties throughout the hospital, analyzing clinical breastfeeding charts for difficult breastfeeding problems, and providing nursing visits and consultations to promote and facilitate breastfeeding in preterm infants.

Personalized and breastfeeding techniques must analyze the receptivity among mothers of preterm infants to various types of breastfeeding information. The written breastfeeding information has been widely used in health education in recent years, and is now being used to provide the mothers of preterm infant with instructions on feeding and holding positions during mother-infant separation. Breastfeeding workshops that include questionnaires, experience, and story sharing can help to keep interest in breastfeeding alive. With the advances in internet technology, almost all mothers of preterm infants have used their cellphones to collect breastfeeding information. Scanning QR codes to deliver breastfeeding information in the form of small videos, microfilms, animations, and short science articles can also provide a more vivid and intuitive understanding for mothers of preterm infants and family members.

Increased behavioral and emotional support come from a variety of sources, including family and medical personnel. We discovered through interviews that most mothers of preterm infants believed that behavioral and emotional support from a variety of sources would help them cope with parenting stress more effectively to support breastfeeding. Maternal social support is positively associated with breastfeeding self-efficacy [46, 47]. The family is the most essential link in the social support network, and family support has a greater influence on breastfeeding rates [48]. Breastfeeding promotion and education at all stages is not only aimed at mothers of preterm infants, but also other family members [49]. Besides, some studies indicate that providing milk for their preterm infants helps mothers cope with the emotional stresses surrounding the neonatal ward experience and gives them a tangible claim on their infants [24, 50].

### Strengths and limitations of the study

This study provided new information regarding breastfeeding motivation, breastfeeding challenges, breastfeeding support, and response to parental stress among mothers separated from their preterm infants at a hospital in Shanghai. This study used primary data using a qualitative approach that should be considered a study strength. However, only the mothers of preterm infants were recruited. Family members are also essential to promote breastfeeding. It is recommended that in future studies perceptions of family members on maternal breastfeeding should be considered.

## Conclusion

In conclusion, breastfeeding was strongly associated with the growth and development of preterm infants. The mothers of preterm infants had a strong desire to breastfeed, but when preterm infants were discharged from the hospital, they encountered many breastfeeding challenges. The mothers of preterm infants lack breastfeeding knowledge and access to knowledge, and they are stressed during the breastfeeding process. Public breastfeeding activity centers for preterm newborns and community breastfeeding support groups for preterm infants are currently being established in China to maintain breastfeeding for preterm infants. Moreover, policies to protect, promote, and support breastfeeding should be drafted and agreed upon by a multidisciplinary healthcare team, including physicians, nurses, midwives, lactation consultants, facility management, and parents (especially husbands).

### Electronic supplementary material

Below is the link to the electronic supplementary material.

Supplementary Material 1

## Data Availability

The datasets generated and/or analysed during the current study are in Chinese language and are not publicly available due to confidentiality of the participants, but are available from the corresponding author on reasonable request.
